# Preferences and Willingness to Pay for Smart Bracelets Among Chinese Pregnant and Postpartum Women: Discrete Choice Experiment

**DOI:** 10.2196/55941

**Published:** 2026-05-12

**Authors:** Jing Wang, Mingyue Cheng, Jialiang Feng, Wenzhuo Li, Xiaotao Zhou, Yufang Cao, Haiyan Feng, Fan Yang, Xiaoyun Wang, Yibo Wu

**Affiliations:** 1 Department of Nursing, the Fourth Affiliated Hospital of School of Medicine, and International School of Medicine, International Institutes of Medicine, Zhejiang University, N1 Shangcheng Avenue, Yiwu, Zhejiang Province, China, 86 18810169630; 2 School of Public Health, Peking University, Beijing, China; 3 Department of Nursing, Inner Mongolia Maternity and Child Health Care Hospital, Hohhot, China; 4 Zhongnan Hospital, Wuhan University, Wuhan, Hubei, China; 5 Medical College, Qingdao University, Qingdao, Shandong, China; 6 Sinopec Zhongyuan Petroleum Engineering Co. Ltd. Tarim Branch Company,Bayingolin Mongolian Autonomous Prefecture, Xinjiang, China; 7 Department of Obstetrics, Inner Mongolia Maternity and Child Health Care Hospital, Hohhot, China; 8 Party Committee Office, Inner Mongolia Maternity and Child Health Care Hospital, Hohhot, China

**Keywords:** pregnant and postpartum women, smart bracelets, discrete choice experiment, willingness to pay, women’s health

## Abstract

**Background:**

Pregnant and postpartum women encounter various health challenges, including physiological stress and mental health issues, which necessitate ongoing health monitoring. Smart bracelets present a promising solution; however, there is limited research on the preferences and willingness to pay (WTP) for such devices among this demographic.

**Objective:**

This study aimed to investigate the preferences and WTP for smart bracelet attributes among pregnant and postpartum women in China and to explore how these preferences vary by sociodemographic factors, pregnancy stage, parity, and complications.

**Methods:**

A cross-sectional discrete choice experiment (DCE) was conducted involving 464 pregnant and postpartum women recruited from a maternal and child health hospital in Inner Mongolia. Six key attributes were evaluated: cost, hospital backend monitoring, primary function, privacy protection, ease of use, and monitoring report frequency. A mixed logit model was used to estimate preference weights and WTP for each attribute, with subgroup analyses based on income, employment, gestational stage, parity, and other factors.

**Results:**

Among the 464 pregnant and postpartum women included in the final analysis (valid data rate: 96.67%), the mean age was 31.06 (SD 4.05) years. The majority of participants were of Han ethnicity (n=385, 82.97%), had a high level of education (n=422, 90.95%), resided in urban areas (n=446, 96.12%), and were employed (n=353, 76.08%). In the DCE, cost negatively impacted smart wristband preferences (β=−0.000257; *P*=.01). Participants exhibited a strong preference for wristbands with fetal heart monitoring (β=1.275; *P*<.001), high-level privacy protection (β=.541; *P*<.001), and ease of use (β=.973; *P*<.001). They were willing to pay ¥4967.45 (based on an exchange rate of US $1=CN ¥6.93) for fetal heart monitoring, ¥2975.17 for sleep monitoring, ¥2109.29 for high-level privacy protection, and ¥3437.09 for daily monitoring. Subgroup analyses indicated that preferences varied according to income, employment, pregnancy stage, parity, complications, and age.

**Conclusions:**

The design of smart bracelets should be tailored to meet the diverse needs of pregnant and postpartum users. Key considerations include the integration of fetal heart and vital sign monitoring, the assurance of data privacy, the enhancement of usability, and the provision of cost-effective options. Understanding the specific preferences of different subgroups can guide the development of inclusive and responsive wearable health technologies for maternal care.

## Introduction

The global proportion of pregnant women who are older, multiparous, or have underlying health conditions is increasing, leading to heightened risks during childbirth and a higher incidence of birth defects in their offspring [1]. According to the World Health Organization (WHO), approximately 280,000 women die each year due to complications related to pregnancy and childbirth, with 90% of these maternal deaths occurring in low- and middle-income countries [2]. Furthermore, pregnant women encounter mental health challenges, such as anxiety and depression [3], both during pregnancy and postpartum, which necessitate timely attention and intervention. With the evolving population structure in China and growing awareness of health management, pregnancy-related health monitoring has emerged as a significant public health issue [4]. Pregnant and postpartum women face various challenges, including physiological changes, increased metabolic demands, and escalating mental health risks, which require precise and continuous health data support [5,6]. While traditional pregnancy management methods are important, they have limitations regarding real-time data, personalization, and continuity [7-9].

With the rapid development of mobile internet and wearable technologies, smart wristbands have emerged as promising tools for convenient and real-time health monitoring in maternal health management. Recent studies indicate that wearable sensors offer a noninvasive approach to monitor pregnancy-related physiological and behavioral changes, facilitating continuous tracking of maternal heart rate, activity, and sleep patterns. These parameters are closely associated with pregnancy-related hormonal fluctuations, thus providing potential tools for the early detection of adverse outcomes [10]. Furthermore, the integration of wearable sensors with artificial intelligence (AI)–assisted data processing techniques is increasingly being explored for pregnancy monitoring. This integration captures multimodal signals, including electrocardiograms, uterine contractions, and fetal movements, thereby enhancing early warning systems and personalized management throughout gestation [11]. A diverse range of wearable technologies, from bracelets and smartwatches to innovative remote home monitoring systems, are being assessed for continuous remote obstetric care. While these technologies demonstrate feasibility, they also underscore the necessity of addressing challenges such as user compliance and data reliability [12].

Compared to traditional methods, smart wristbands offer significant advantages in maternal health management. They facilitate continuous monitoring of physiological data, such as heart rate, activity levels, and sleep quality [11], and can even perform fetal heart monitoring [13]. This capability provides personalized health management solutions for pregnant women, minimizing the need for frequent hospital visits, thereby saving both time and effort [14]. In terms of real-time monitoring, the data collected by smart wristbands can be instantly synchronized to smartphones or the cloud, enabling pregnant women and their health care providers to monitor health status in real time and promptly identify potential issues. For instance, a study by Grym et al [15] assessed the feasibility of continuous health parameter monitoring via smart wristbands during pregnancy and 1 month postpartum. The findings indicated that smart wristbands effectively collected data on activity, sleep, and heart rate, thus offering personalized health management solutions for pregnant women. However, the study also highlighted that comfort and data synchronization issues could hinder sustained use. Furthermore, Alim and Imtiaz [13] conducted a systematic review analyzing the use of various wearable sensors in maternal health monitoring, concluding that these devices could effectively track fetal electrocardiograms, fetal movement, and maternal physical activity, contributing to the reduction of pregnancy-related risks.

Despite the growing market for smart wristbands, research on the specific preferences and willingness to pay (WTP) for features, design, and price points among pregnant women remains limited. Understanding the actual needs of this population, as well as their acceptable price levels, is crucial for the functional design, market promotion, and optimization of health management services related to smart wristbands. Existing studies have primarily focused on technical feasibility, device accuracy, and user acceptance while offering limited in-depth analysis of pregnant women’s preferences for specific features and their WTP. For instance, Wu et al [16], using the Technology Acceptance Model, evaluated pregnant women’s willingness to use smart fetal heart monitoring devices, revealing that perceived usefulness and ease of use significantly influenced their willingness to adopt such technology. However, this study did not explore specific feature preferences or price sensitivity. Additionally, Schramm et al [17] examined the acceptance of noninvasive electronic devices for pregnancy monitoring among 507 pregnant women, concluding that women were generally open to using these devices for monitoring purposes. Nevertheless, these studies mainly concentrated on preliminary assessments of technology acceptance and WTP, lacking detailed analyses of pregnant women’s preferences for specific features (eg, fetal heart monitoring and sleep quality assessment), hospital backend monitoring, and report frequency. Beyond device features, factors such as price, ease of operation, and privacy protection also significantly influence pregnant women’s willingness to use smart wristbands [16,18]. Elevated prices may deter potential purchasers, while complex interfaces can detrimentally affect user experience. Therefore, the design of smart wristbands should thoroughly address these needs and offer more personalized and comprehensive services.

The functional needs of pregnant women for smart wristbands vary significantly across different stages of pregnancy. In the early stages, women are primarily concerned with features that alleviate morning sickness and enhance sleep quality [19]. As pregnancy progresses into the midstage, with fetal development stabilizing, there is an increased demand for activity monitoring and weight management [20]. In the later stages, as childbirth approaches, women focus more on fetal heart monitoring, uterine contraction tracking, and other functionalities that provide insights into fetal health [21]. After childbirth, new mothers particularly seek features for sleep monitoring, guidance on physical recovery, and parenting resources to help them adapt to their new lifestyle [22-24]. Consequently, the design of smart wristbands must comprehensively address the distinct needs at each stage of pregnancy, offering targeted health management solutions.

Discrete choice experiments (DCEs) are a crucial preference measurement tool extensively used in the health care sector, particularly in maternal and child health management. Through DCEs, researchers can gain profound insights into the preferences of patients and the public regarding various medical interventions, service formats, and policy designs, thereby providing a solid foundation for decision-making. For instance, in drug selection, DCEs can elucidate how patients prioritize key attributes such as drug efficacy, side effects, mode of administration, and cost, thereby offering valuable guidance for drug development and market promotion. Arije et al [25] discovered that adolescents and young individuals in Nigeria exhibited diverse preferences for sexual and reproductive health services, emphasizing factors such as the medical environment, physician professionalism, service attitudes, medical expenses, and waiting times. Furthermore, DCE plays a pivotal role in health policy evaluation. Luo et al [26] used DCE to evaluate the preferences of public health master’s graduates regarding job positions, revealing that monthly salary was the most significant determinant influencing their employment choices, followed by opportunities for career advancement and job location. Donnan et al [27] found that consumers expressed heightened concerns regarding the regulatory status of e-cigarettes and demonstrated a WTP premium prices for products regulated by Canada’s Ministry of Health. Additionally, Jones et al [28] used DCE to generate health utility values for patients with chronic obstructive pulmonary disease and explored its application in Health Technology Assessment. These studies underscore the extensive potential of DCE in understanding patient and public needs and in formulating effective health policies and interventions.

This study aimed to use DCEs to systematically evaluate the preferences of pregnant women regarding the attributes of smart wristbands and to estimate the additional amount they are willing to pay for these attributes. The findings will offer crucial insights for decision-making among smart wristband manufacturers, health service providers, and policymakers, thereby promoting the development of smart wristband products and services that are better aligned with the needs of pregnant women. This alignment is expected to improve maternal health management and ultimately enhance maternal and child health outcomes.

## Methods

### Study Design

This study aimed to explore the preferences and WTP for smart bracelets among pregnant and postpartum women using a DCE. The participants consisted of women who were pregnant or had given birth within the past 2 years, with a sample randomly selected from the Inner Mongolia Maternal and Child Health Hospital. Demographic diversity was intentionally incorporated into the sample to ensure that the research findings are broadly applicable to various subgroups of pregnant and postpartum women in the region. Using the DCE method, the study systematically assessed the preferences of pregnant and postpartum women for smart bracelets, focusing on the device’s functionality, price, and other design features, while examining their WTP for different combinations of product features. This approach provided valuable insights into their preference patterns and market demand.

This was a cross-sectional study designed to collect data in a single survey, assessing the preferences and WTP for smart bracelets among pregnant and postpartum women. To ensure the accuracy and feasibility of the study, a comprehensive set of inclusion and exclusion criteria was established.

### Inclusion Criteria

The inclusion criteria were as follows: (1) age ≥18 years, (2) female, (3) Chinese nationality (People’s Republic of China), (4) pregnant or had given birth within the past 2 years, (5) able to complete the online survey independently or with the assistance of the investigator, (6) having basic literacy skills and being able to communicate and interact normally, and (7) willing to voluntarily participate in the study and provide informed consent.

### Exclusion Criteria

The exclusion criteria were as follows: (1) individuals who were unconscious or had mental abnormalities, (2) individuals with cognitive impairments, (3) individuals currently participating in other similar research studies, (4) individuals unwilling to cooperate, and (5) individuals with other severe health conditions that may affect the study results.

Through this approach, the study provided valuable market and preference data for the use of smart bracelets among pregnant and postpartum women, aiding in the development and promotion of related products.

### Ethical Considerations

The study received approval from the Ethics Committee of Inner Mongolia Maternal and Child Health Hospital (review [176], 2024). All participants voluntarily engaged in the study and provided informed consent, confirming their understanding of the study’s objectives, methods, and potential risks. This consent was obtained prior to their participation, adhering to ethical standards for research involving human subjects. Given the involvement of human participants, appropriate ethical oversight was ensured, with all research activities sanctioned by the Ethics Committee. To safeguard privacy and confidentiality, all study data were anonymized and deidentified, thereby protecting participants’ identities. The data were securely stored and accessible only to authorized personnel involved in the research. No compensation was offered to participants for their involvement in this study. To further uphold the confidentiality and anonymity of participants, no identifiable images or data of individual participants were included in the manuscript or supplementary materials.

### Development of DCE

In this study, we conducted a systematic review to comprehensively identify and assess the key attributes and their levels that pregnant and postpartum women prioritize when selecting a smart wristband. We searched the literature across several key databases, including PubMed, Cochrane Library, Web of Science, CNKI (China National Knowledge Infrastructure), and Scopus, to ensure thorough coverage of relevant research on smart wristbands and wearable devices in maternal health management. A combination of Medical Subject Headings (MeSH) and free-text keywords was used to filter the relevant literature. The search terms included “wearable devices,” “smart wristband,” “fitness tracker,” “wearable sensors,” “maternal health,” “pregnancy,” “pregnant women,” “postpartum,” and “remote monitoring,” along with various combinations (eg, “wearable devices AND pregnancy,” “smart wristband AND maternal health”). These terms were tailored to each database’s search syntax to ensure both sensitivity and specificity in retrieval.

The literature was screened based on preestablished inclusion criteria: the research subjects had to be either pregnant or postpartum women or related health research; the studies needed to involve smart wristbands or other wearable devices; and relevant attributes such as price, privacy protection, or functionality had to be mentioned in the literature. Initially, all retrieved records were screened by title and abstract, followed by a full-text review of potentially eligible papers to confirm their eligibility.

This systematic review encompassed 7 highly relevant studies [13,29-34]. Through analysis of these studies, the research team identified 6 key attributes that pregnant and postpartum women prioritize when selecting a smart wristband: price, hospital background monitoring management, main function, privacy protection, ease of use, and frequency of monitoring report delivery. The levels associated with each of these attributes were also extracted from the literature, establishing a foundation for subsequent expert consultation.

Following the systematic review, expert consultations were conducted to further validate and refine the identified attributes and levels. These consultations involved obstetricians with clinical experience in prenatal and postpartum care (3 experts), health management professionals knowledgeable about maternal health service delivery (2 experts), wearable technology specialists with expertise in product design and human factors (2 experts), and market research experts experienced in consumer preference studies (2 experts). In total, 9 experts participated in 2 rounds of consultation.

During the first round, experts reviewed the preliminary list of attributes and levels. They were asked to comment on the relevance of each attribute, suggest modifications to levels (eg, cost ranges and descriptions of functionality), and propose any missing attributes. Specific expert feedback included the following:

- Cost: The technology and market experts suggested that cost levels should represent realistic price points in the current Chinese wearable market for consumer smart wristbands targeted at health care use (eg, entry level, midrange, and high end). As a result, the cost levels were adjusted to ¥250, ¥500, ¥750, and ¥1000 after consensus.
- Hospital background monitoring management: Experts highlighted that integration with hospital management systems may be crucial for clinical acceptance by pregnant women but that such integration is not universally available. On the basis of this feedback, the level description was refined to clearly distinguish between “yes (supported by hospital monitoring)” and “no (standalone device).”
- Main function: Obstetrics and technology experts emphasized including specific vital sign monitoring relevant to pregnancy (eg, blood pressure and heart rate) and fetal heart monitoring—features frequently mentioned in the literature as highly valued by users. Therefore, levels were expanded to include “activity tracking,” “vital sign monitoring,” “fetal heart monitoring,” and “sleep quality.”
- Privacy protection: Health management and privacy experts recommended distinguishing among degrees of data protection (high, standard, and none) to reflect different security protocols and possible user concerns highlighted in digital health studies.
- Ease of use: Experts underscored the importance of usability, especially for pregnant women with varying levels of technology literacy. The levels were refined to a 4-tier scale, ranging from “very easy to use” to “very difficult to use.”
- Frequency of monitoring report delivery: Feedback from both market and clinical experts suggested that delivery frequency might influence perceived utility; levels were defined to cover daily to weekly reporting patterns relevant to routine maternal health monitoring.

After incorporating these suggestions, a second consultation round confirmed that the refined attributes and levels were appropriate, meaningful, and aligned with the decision context and target population’s concerns. Experts agreed that the defined levels reflected realistic choices that pregnant and postpartum women would encounter when choosing a smart wristband.

Through the systematic review and expert consultation, the 6 key attributes and their levels, along with the definitions of each attribute, that pregnant and postpartum women focus on when selecting a smart wristband were ultimately determined (Table 1).

**Table 1.** Attributes and levels of the smart bracelet.

Attribute, definition, and levels	Level explanation
Cost (CN ¥)^a^: refers to the amount of money that a user needs to pay to purchase the smart wristband.	
250	Entry-level price point representing basic device functionality.
500	Midlower price point with a balance of features and affordability.
750	Midhigher price point offering more advanced features.
1000	High-end price point with comprehensive functions and potential premium services.
Hospital background monitoring management: indicates whether the smart wristband supports integration with a hospital’s or health care provider’s monitoring and management system.	
Yes	Device can securely transmit user health data to a linked clinical or hospital system for professional review, enabling clinician follow-up and intervention.
No	Device operates independently without connection to a hospital or clinical system; users cannot share data directly with clinicians.
Main function: describes the core monitoring or tracking capabilities of the smart wristband.	
Activity tracking	Tracks basic physical activity such as step count and movement patterns.
Vital sign monitoring	Measures critical health indicators such as heart rate and blood pressure.
Fetal heart monitoring	Continuously records fetal heart rate patterns relevant to pregnancy monitoring.
Sleep quality	Monitors sleep stages and sleep quality metrics such as duration and restfulness.
Privacy protection: refers to the level of data security and privacy safeguards implemented by the device during data collection, storage, and transmission.	
High-level protection	Device uses strong privacy measures such as end-to-end encryption, strict access control, no third-party data sharing, and adherence to recognized privacy standards to protect highly sensitive health data.
Standard protection	Device applies basic encryption and authentication methods to protect data, with typical privacy policy terms and moderate risk mitigation.
No special protection	Device lacks dedicated privacy or security mechanisms; data may be stored or transmitted without encryption or user-controlled safeguards.
Ease of use: describes how simple or complex the device is for users to operate and interact with.	
Very easy to use	Interface and workflows are intuitive, requiring minimal steps and no user training.
Relatively easy to use	Most tasks are straightforward, with occasional need for guidance.
Relatively difficult to use	Multiple steps or unclear interface elements may cause confusion for some users.
Very difficult to use	Complex interactions and navigation require frequent assistance or training.
Frequency of monitoring report delivery to the smart bracelet: indicates how often the user receives summary reports or actionable insights based on the device’s monitoring data.	
Once a day	Daily reporting frequency.
Once every 2 days	Reporting every second day.
Once a week or less often	Weekly or longer intervals between reports.

a A currency exchange rate of US $1=CN ¥6.93 was applicable.

On the basis of the attributes and levels determined earlier, our team used a fractional factorial design method to determine the optimal number of attributes that pregnant women prefer when selecting a smart bracelet. Given that the 6 attributes in this study each contain 2 to 4 levels, a full factorial design would result in 1152 combinations (4×2×4×3×4×3=1152), which is clearly impractical to generate. Therefore, we used a fractional factorial design to determine the optimal number of choice sets. This method is based on 2 principles: the orthogonality principle and the balance principle [35,36]. The choice sets are generated using orthogonal experiments in SPSS AU software (Beijing Qingsi Technology Co). An orthogonal strategy is selected based on the orthogonal table manual, and ineffective designs are eliminated through internal validity checks, leading to the final design. Suitable options were selected as controls to form multiple-choice sets. Each participant faced 12 choice sets, with each choice set containing 3 options: smart bracelet A, smart bracelet B, and “none.” To assess the validity of the DCE questionnaire, a choice set with a clear dominant option (a lie detector question) was included in the questionnaire. Only when participants select the correct option from this set will their data be included in the subsequent analysis. Meanwhile, according to the empirical rule proposed by Johnson and Orme [37,38], the minimum sample size for a DCE study can be calculated using the formula:


N§gt;500×c / (t×a)

where N is the minimum sample size, representing the number of participants required in the study; c is the largest number of levels for any attribute (c=4), t is the number of choice tasks (*t*=12), and a is the number of alternatives (a=2).

The final calculation resulted in a minimum sample size of 84.

Table 2 is an example of a DCE choice set.

**Table 2.** Example of a discrete choice experiment choice set.

Attribute	Smart bracelet A	Smart bracelet B	None of them
Cost (¥)^a^	1000	500	Choose none of them
Hospital background monitoring management	No	Yes	—^b^
Main function	Activity tracking	Fetal heart monitoring	—
Privacy protection	High-level protection	High-level protection	—
Ease of use	Relatively difficult to use	Very easy to use	—
Frequency of monitoring report delivery to the smart bracelet	Once a day	Once every 2 days	—

a A currency exchange rate of US $1=CN ¥6.93 was applicable.

b Not applicable.

### Data Collection

Data were collected using an online survey platform, such as WenjuanXing. The study commenced in September 2024 and concluded in May 2025, lasting approximately 9 months. Recruitment took place from February 2025 to April 2025. The data collection method involved researchers identifying eligible participants offline and requesting them to scan a WenjuanXing link to complete the online survey. During the recruitment period, researchers provided eligible pregnant and postpartum women with comprehensive information about the study and invited them to participate by completing the online questionnaire.

The survey was divided into 2 distinct parts. The first part gathered essential demographic information from participants, including age, education level, and place of residence, to gain a comprehensive understanding of their backgrounds. The second part focused on the preferences of pregnant and postpartum women concerning smart bracelets, using the DCE method for data collection. The DCE questionnaire featured multiple-choice scenarios, each presenting 2 different combinations of smart bracelet attributes, from which participants were asked to select the combination that best aligned with their preferences. The attributes of the smart bracelets encompassed device functions (such as heart rate monitoring, activity tracking, and sleep tracking), price, ease of use, and privacy protection, all chosen based on prevalent features available in the current market. To mitigate sequence effects, all choice scenarios were randomly arranged.

To ensure data quality and protect participants’ privacy, the survey was conducted anonymously, using encryption technology to safeguard the data. All participants voluntarily completed the questionnaire after receiving an informed consent form, which ensured their understanding of the study’s purpose and their involvement [39]. Researchers provided necessary assistance to facilitate the completion of the survey. During data collection, logical checks and additional measures were implemented to ensure the validity and consistency of the data.

### Statistics

Descriptive statistics were used to summarize the sociodemographic characteristics of the participants, including age, income, education, occupation, pregnancy stage, parity, and the presence of pregnancy complications. Means and SDs were computed for continuous variables, while frequencies and percentages were reported for categorical variables. To analyze participants’ preferences regarding smart bracelet attributes, a mixed logit model was used. This model accommodated preference heterogeneity among individuals by allowing the attribute coefficients to vary randomly. Each participant’s utility for a specific smart bracelet profile was modeled as a linear combination of attribute levels. The estimated parameters included the fixed effects (β) of each attribute level, their SEs, and corresponding *P* values. The relative importance scores quantify the significance of each attribute, calculated by dividing the difference in utility between the lowest and highest levels of that attribute by the sum of the differences for all attributes. Cost was treated as a continuous variable to facilitate the estimation of participants’ WTP for different attributes. WTP values were derived by dividing the attribute coefficient by the negative of the cost coefficient, reflecting the monetary value participants assigned to specific smart bracelet features. Subgroup analyses were conducted to investigate differences in preferences and WTP across various demographic and clinical subgroups, including income levels, employment status, pregnancy stages, parity, pregnancy complications, and age. All statistical analyses were executed using Stata 15.0 (StataCorp LLC). A 2-sided *P* value of <.05 was deemed statistically significant.

## Results

### Characteristics of Respondents

A total of 480 pregnant women were initially recruited for the study; after excluding those with incomplete data or failing logical consistency checks, 464 participants were included in the final analysis, yielding a high data validity rate of 96.67%. Among the 464 participants, the mean age was 31.06 (SD 4.05) years (Table 3). The majority were of Han ethnicity (385, 82.97%) compared to ethnic minorities (n=79, 17.03%). For education level, 90.95% (n=422) had a high level of education. Most participants resided in urban areas (n=446, 96.12%). Income distribution was as follows: ≤4000 (n=102, 21.98%), 4001 to 6000 (n=170, 36.64%), and ≥6001 (n=192, 41.38%). Occupational status included employed (n=353, 76.08%), unemployed or out of work (n=60, 12.93%), and without fixed occupation (freelance, n=51, 10.99%). Pregnancy status was distributed as early pregnancy (1‐13 wk; n=122, 26.29%), midpregnancy (14‐27 wk; n=107, 23.06%), late pregnancy (≥28 wk; n=110, 23.71%), and postpartum (n=125, 26.94%). First-time pregnancy was reported by 71.98% (n=334). Regarding pregnancy complications, 84.05% (n=390) had none.

**Table 3.** Demographic characteristics of maternal (N=464).

Variables	Values
Age (y), mean (SD)	31.06 (4.05)
Ethnicity, n (%)	
Han ethnicity	385 (82.97)
Ethnic minorities	79 (17.03)
Education level, n (%)	
Primary and middle education	42 (9.05)
High education	422 (90.95)
Place of residence, n (%)	
Urban	446 (96.12)
Rural	18 (3.88)
Income, n (%)	
≤4000	102 (21.98)
4001‐6000	170 (36.64)
≥6001	192 (41.38)
Occupation, n (%)	
Employed	353 (76.08)
No fixed occupation (freelancer)	51 (10.99)
Unemployed or out of work	60 (12.93)
Pregnancy period, n (%)	
Early pregnancy	122 (26.29)
Midpregnancy	107 (23.06)
Late pregnancy	110 (23.71)
Postpartum	125 (26.94)
First childbirth, n (%)	
Yes	334 (71.98)
No	130 (28.02)
Pregnancy complications, n (%)	
Yes	74 (15.95)
No	390 (84.05)

### DCE Results

This study analyzed the preferences and WTP of pregnant and postpartum women for smart wristbands through a DCE (Table 4). The results showed that cost had a negative impact on choice (β=−0.000257; *P*=.01). For the hospital background monitoring management attribute, the most preferred level was having hospital background monitoring management (β=.226; *P*=.03); in terms of primary functions, the most preferred function was fetal heart monitoring (β=1.275; *P*<.001); for the privacy protection attribute, the most preferred level was high-level protection (β=.541; *P*<.001); in terms of ease of use, the most preferred level was relatively easy to use (β=.973; *P*<.001); and for the monitoring report frequency, the most preferred level was once a day (β=.882; *P*<.001).

**Table 4.** Mixed logit model estimates of smart bracelet preferences among pregnant and postpartum women^a^.

Attributes and levels	β (SE)	95% CI	*P* value
Cost	−0.000257 (0.000099)	−0.000451 to −0.000062	.01
Hospital background monitoring management			
No	Ref^b^	—^c^	—
Yes	0.226 (0.105)	0.020 to 0.431	.03
Main function			
Activity tracking	Ref	—	—
Sleep quality	0.763 (0.074)	0.619 to 0.908	<.001
Vital sign monitoring	1.233 (0.114)	1.010 to 1.457	<.001
Fetal heart monitoring	1.275 (0.087)	1.104 to 1.446	<.001
Privacy protection			
No special protection	Ref	—	—
Standard protection	0.218 (0.083)	0.054 to 0.381	.009
High-level protection	0.541 (0.104)	0.338 to 0.745	<.001
Ease of use			
Very difficult to use	Ref	—	—
Relatively difficult to use	0.662 (0.117)	0.433 to 0.892	<.001
Very easy to use	0.719 (0.132)	0.460 to 0.978	<.001
Relatively easy to use	0.973 (0.094)	0.788 to 1.158	<.001
Frequency of monitoring report delivery			
Once a week or less often	Ref	—	—
Once every 2 days	0.571 (0.132)	0.312 to 0.830	<.001
Once a day	0.882 (0.109)	0.669 to 1.095	<.001

a Observations=16,704; Log Likelihood (null)=−4486.437; Log Likelihood (model)=−4386.441; df=23; Akaike Information Criterion=8818.882; Bayesian Information Criterion=8996.521.

b Ref: reference.

c Not applicable.

### The Percent Importance of Smart Wristband Attributes

As presented in Table 5, the main function was the most important attribute.

**Table 5.** Importance of attributes.

Attribute	Importance (%)
Cost	0.01
Hospital background monitoring management	5.80
Main function	32.72
Privacy protection	13.88
Ease of use	24.97
Frequency of monitoring report delivery to the smart bracelet	22.63

### WTP Results

Table 6 presents the estimated WTP for changes in specific attributes of smart bracelets, with WTP values quantifying participants’ implicit monetary trade-offs between improvements in these attributes. In DCE studies, WTP reflects the marginal additional amount a respondent is hypothetically willing to pay for an enhancement in a given attribute level relative to a reference level. This is derived from the ratio of attribute coefficients to the cost coefficient in the mixed logit model, effectively capturing the relative valuation of attribute changes. Compared to the reference level of activity tracking, participants were willing to pay an additional ¥2975.168 (95% CI 743.556-5206.781) for sleep quality, ¥4806.745 (95% CI 990.014, 8623.475) for vital sign monitoring, and ¥4967.454 (95% CI 1212.358, 8722.550) for fetal heart monitoring. For the privacy protection attribute, participants were willing to pay ¥847.659 (95% CI 162.234-1533.085) for standard protection and ¥2109.294 (95% CI 551.576-3667.013) for high-level protection, both compared to no special protection. Regarding ease of use, relative to the category of “very difficult to use,” participants were willing to pay ¥2581.683 (95% CI 598.881-4564.485) for relatively difficult-to-use devices, ¥2802.522 (95% CI 539.744-5065.300) for devices that are very easy to use, and ¥3792.292 (95% CI 860.861-6723.723) for relatively easy-to-use devices. Finally, concerning monitoring frequency, compared to the reference level of “once a week or less often,” participants were willing to pay ¥2225.933 (95% CI 148.084-4303.781) for using it once every 2 days and ¥3437.094 (95% CI 713.160-6161.028) for using the device once a day.

**Table 6.** Estimated willingness to pay (WTP) for each attribute level.

Attributes and levels	WTP (95% CI), ¥^a^
Hospital background monitoring management	
No	Ref^b^
Yes	878.803 (−84.138 to 1841.745)
Main function	
Activity tracking	Ref
Sleep quality	2975.168 (743.556 to 5206.781)
Vital sign monitoring	4806.745 (990.014 to 8623.475)
Fetal heart monitoring	4967.454 (1212.358 to 8722.550)
Privacy protection	
No special protection	Ref
Standard protection	847.659 (162.234 to 1533.085)
High-level protection	2109.294 (551.576 to 3667.013)
Ease of use	
Very difficult to use	Ref
Relatively difficult to use	2581.683 (598.881 to 4564.485)
Very easy to use	2802.522 (539.744 to 5065.300)
Relatively easy to use	3792.292 (860.861 to 6723.723)
Frequency of monitoring report delivery	
Once a week or less often	Ref
Once every 2 days	2225.933 (148.084 to 4303.781)
Once a day	3437.094 (713.160 to 6161.028)

a A currency exchange rate of US $1=CN ¥6.93 was applicable.

b Ref: reference.

### Subgroup Results

We further conducted subgroup analyses based on demographic and clinical characteristics, including income, employment status, pregnancy stage, parity, presence of pregnancy complications, and age (Tables S1-S7 in Multimedia Appendix 1). The results showed that these characteristics significantly influenced pregnant and postpartum women’s preferences for the attributes of smart bracelets. In terms of the price attribute, pregnant and postpartum women with low income (income ≤ ¥4000), those who were unemployed, those in early or late pregnancy, those with previous births, those without pregnancy complications, and those of younger age (age ≤ 30 y) exhibited a significantly stronger preference for lower-priced smart bracelets (*P*<.05); whereas women with moderate income (¥4001‐¥6000), those without fixed employment (self-employed), primiparous women, those without pregnancy complications, and younger women showed a significantly stronger preference for smart bracelets with hospital background monitoring functions (*P*<.05). In terms of specific feature preferences, pregnant and postpartum women with low and high income, those who were employed, those in mid to late pregnancy, both primiparous and multiparous women, women with or without pregnancy complications, and older women (age >30 y) exhibited a significant preference for smart bracelets equipped with fetal heart monitoring functions (*P*<.05), whereas women with moderate income, those without fixed employment, and those who were unemployed, women in early pregnancy or postpartum, and younger women exhibited a significant preference for smart bracelets with vital sign monitoring functions (*P*<.05). Except for unemployed pregnant and postpartum women, all other subgroups showed a significant preference for smart bracelets with high levels of privacy protection (*P*<.05). With respect to ease of use, pregnant and postpartum women without fixed employment and those in late pregnancy showed a stronger preference for very easy-to-use devices, while the remaining subgroups all preferred devices that were relatively easy to use (*P*<.05). In addition, all subgroups of pregnant and postpartum women exhibited a significant preference for a once-daily notification frequency. Overall, factors such as income, employment status, pregnancy stage, parity, presence of pregnancy complications, and age influenced Chinese pregnant and postpartum women’s preferences for different smart bracelet attributes to varying degrees.

## Discussion

### Principal Findings

Our results indicate that the functionality of smart bracelets is the most influential attribute affecting participant preferences, followed by ease of use, frequency of monitoring reports, and price. Participants favored smart bracelets that were affordably priced, offered hospital backend monitoring, included fetal heart rate monitoring, ensured a high level of privacy protection, were relatively user-friendly, and provided frequent monitoring reports. Furthermore, our findings reveal that participant preferences for smart bracelets varied based on sociodemographic characteristics, gestational stage, parity (first-time pregnancy), and the presence of pregnancy complications. Understanding the preferences of participants within these subgroups may facilitate the development of tailored smart bracelets.

The preference for lower-cost smart bracelets aligns with previous research [40]. Our subgroup analysis further demonstrated that participants with lower incomes and those who were unemployed exhibited a significant preference for lower-priced smart bracelets. Jacob et al [41] found that low-income individuals display greater price sensitivity compared to their high-income counterparts, a sensitivity that is exacerbated by income disruption among the unemployed. Additionally, we observed that participants in both early and late pregnancy showed a stronger inclination toward lower-priced smart bracelets. The increased price sensitivity among women in early pregnancy may be attributed to their anticipation of forthcoming childbirth-related expenses, such as delivery fees and newborn care [42]. Notably, heightened price sensitivity during late pregnancy could be linked to the expectation of additional costs related to the imminent delivery [43]. Furthermore, our study revealed that participants who were not experiencing their first pregnancy also exhibited heightened price sensitivity, potentially due to the stress of reallocating household resources [44]. Younger pregnant individuals demonstrated a higher level of price sensitivity, likely reflecting their position in the early stages of their careers, where income levels are generally lower compared to those in mid-to-late career stages. This underscores the necessity of providing cost-effective smart bracelets, particularly for lower-income, unemployed, and first-time pregnant individuals.

Participants expressed a clear preference for smart bracelets equipped with hospital backend monitoring. This finding aligns with Bachiri’s research on the integration of mobile health systems, which demonstrated an increase in maternal adherence when hospital data connectivity was enabled [45]. Subgroup analysis revealed that participants with moderate incomes and those experiencing their first pregnancy exhibited a significant preference for hospital backend monitoring, likely due to their reliance on medical authority [46,47]. Additionally, self-employed participants showed a heightened preference for hospital backend monitoring, indicating a perceived need for professional medical support in nontraditional work settings [48]. Younger participants also demonstrated a notable preference for hospital backend monitoring, reflecting a reliance on institutionalized medical care within this demographic [49]. Therefore, incorporating hospital backend monitoring into smart bracelets would address the preferences of first-time mothers, self-employed individuals, and younger users, thereby enhancing trust in the device’s medical relevance. For the design of smart bracelets, it is crucial to integrate fetal heart rate monitoring for late-stage pregnancy while also recognizing the importance of vital sign monitoring for early pregnancy and postpartum users.

Regarding the functionality of smart bracelets, participants favored those with fetal heart rate monitoring. This aligns with prior research, such as Ahmed et al [50], which indicated that fetal heart rate monitoring was the significantly preferred feature among pregnant women, directly related to their sensitivity to pregnancy health risks. Other studies have shown that abnormal fetal heart rates are important early warning signs of pregnancy complications, and the continuous monitoring capabilities of smart bracelets can increase the detection rate of anomalies [13,51]. However, subgroup analysis revealed that both low-income and high-income groups preferred fetal heart rate monitoring, while the middle-income group tended to favor vital sign monitoring. This differential preference among the middle-income group may stem from their heightened health management awareness, leading them to favor multidimensional health data integration [52]. This nonlinear relationship may also arise because high-income individuals prioritize fetal safety, while low-income individuals rely on basic monitoring as a substitute for prenatal care, and middle-income individuals seek comprehensive health management [53-55]. Our research also found that freelancers and unemployed participants preferred vital sign monitoring, while employed participants preferred fetal heart rate monitoring. This may be because self-employment or unemployment allows more time to focus on one’s own physiological changes, such as adjusting lifestyles by monitoring sleep quality or stress levels [56]. Unemployed individuals may experience higher health anxiety due to financial pressures, leading them to prefer vital sign data for preventing potential health risks. For employed individuals under work pressure, fetal heart rate monitoring is viewed as a direct indicator of fetal health, enabling quick identification of anomalies and reducing the frequency of medical visits [7,57]. Additionally, employed individuals may have access to professional prenatal care through workplace medical benefits, thereby reducing their need for vital sign monitoring [58,59]. Participants in early pregnancy and postpartum preferred vital sign monitoring, while those in the mid-to-late stages of pregnancy preferred fetal heart rate monitoring. This may be because, during the initial bodily adaptation phase of early pregnancy, women are more concerned with their own physiological changes (eg, morning sickness and fatigue) [60,61] and early warning signs of pregnancy complications (eg, anemia and hypertension) [62,63], and there is an increased need for health monitoring during the recovery period, such as postpartum depression risk, blood pressure fluctuations, and wound healing tracking [64,65]. Literature indicates that continuous vital sign monitoring is feasible within 7 months postpartum [66]. The mid-to-late stages of pregnancy are critical periods for fetal development. Fetal movement becomes regular after 20 weeks, and fetal heart rate monitoring becomes a core indicator for assessing fetal development, directly related to pregnancy complications such as preeclampsia and fetal growth restriction [67-69]. Pregnant women in late pregnancy experience a significant increase in anxiety about fetal safety, and fetal heart rate data can provide immediate psychological reassurance [70].

Regarding privacy protection, participants generally preferred smart bracelets with a high level of privacy protection, indicating that data security is a core concern for users. This finding aligns with existing research highlighting users’ privacy concerns related to wearable health devices [71,72]. Additionally, participants placed a high value on ease of use. Notably, while very easy-to-use bracelets were favored to some extent, the intensity of preference was lower compared to “relatively easy-to-use” bracelets. This may be due to participants feeling that very simple bracelets lack sufficient functionality or intelligent experience, reflecting a synergistic need for functional depth and operational convenience within the pregnant and postpartum population [73]. This observation aligns with human-computer interaction research on wearable devices, where overly simplified interfaces can undermine user trust in data reliability [74,75]. Younger participants preferred vital sign monitoring, while older participants favored fetal heart rate monitoring. Younger pregnant women perceive their pregnancy risk as lower, focusing more on their own health rather than fetal-specific indicators. In contrast, older mothers face a significantly higher incidence of pregnancy complications (eg, gestational hypertension and premature delivery), leading them to consider fetal heart rate monitoring as a necessary risk management tool [16,76]. Therefore, smart bracelet design should prioritize user privacy and ease of use, balancing simplicity with functionality to build trust among all user demographics.

Regarding the frequency of monitoring report delivery, participants generally preferred smart bracelets capable of providing more frequent updates. This preference indicates that regular health data updates are a significant requirement for pregnant and postpartum individuals seeking to manage their health through real-time feedback. However, it is crucial to acknowledge that high-frequency monitoring may increase health anxiety [77]. Therefore, it is advisable to mitigate the false alarm rate through AI-assisted interpretation [78].

The design of smart bracelets must comprehensively consider key factors such as functionality, price, ease of use, privacy protection, and report frequency. This approach is essential for accurately aligning with the diverse preferences of pregnant and postpartum individuals across various gestational stages, income levels, employment statuses, and parity. Priority should be given to integrating functions for monitoring fetal heart rate and vital signs, with differentiated configurations tailored to user characteristics. While ensuring data privacy and operational convenience, the human-computer interaction interface should be optimized to avoid overly simplified functions that could undermine user trust. Furthermore, establishing appropriate monitoring frequencies and incorporating AI-assisted interpretation can enhance the practicality and reassurance of data feedback. Only by addressing these multifaceted needs can smart wearable devices be effectively tailored to meet the demands of the maternal population.

### Limitations

This study has several limitations. First, the sample was primarily drawn from a specific regional population of pregnant and postpartum individuals, which may not fully represent the preferences of users across diverse regions, cultural backgrounds, and health care environments. Second, the data were based on self-reported responses, which are subject to potential biases, such as subjective perception and social desirability, and may not accurately reflect actual user behavior during device usage. Third, although detailed subgroup analyses were conducted, other potentially influential variables—such as digital health literacy and family support levels—were not explored. Additionally, the selection of smart bracelet features was based on current mainstream product configurations, which may change as technology evolves. Furthermore, while a mixed logit model was appropriately used to estimate preferences and accommodate heterogeneity across the overall sample, some individual subgroups (eg, “unemployed or out of work” and “no fixed occupation (freelancer)”) had relatively small sample sizes. Small subgroup counts can increase uncertainty when interpreting subgroup-specific effects or interactions, potentially yielding unstable estimates or larger SEs for those categories. For this reason, findings related to these small subgroups should be interpreted with caution and not overgeneralized. Future research should consider expanding the sample scope and incorporating longitudinal studies and real-world usage data to further validate and enrich the findings, as well as examine additional factors that may influence preferences for wearable health technology.

### Conclusions

In conclusion, preferences for smart bracelets among pregnant individuals are influenced by various factors, including functionality, price sensitivity, ease of use, and privacy concerns. The most significant features identified are the primary function and ease of use. Design considerations should account for the diverse needs across different sociodemographic groups, stages of pregnancy, and potential complications. Specifically, cost-effective options, robust privacy protections, and user-friendly interfaces are essential to ensure widespread adoption. Furthermore, integrating real-time monitoring with AI-assisted feedback can enhance the usability of smart bracelets, addressing concerns related to health anxiety and improving the overall user experience. Ultimately, a personalized and comprehensive approach to smart bracelet design can better support the health and well-being of pregnant women during both their pregnancy and postpartum periods.

## Supplementary material

10.2196/55941 Multimedia Appendix 1 Subgroup analysis results for preference heterogeneity in the discrete choice experiment.

## Abbreviations

AI: artificial intelligence
CNKI: China National Knowledge Infrastructure
DCE: discrete choice experiment
MeSH: medical subject headings
WHO: World Health Organization
WTP: willingness to pay
