# Supplementary material for: Preferences and Willingness to Pay for Smart Bracelets Among Chinese Pregnant and Postpartum Women: Discrete Choice Experiment
Source: J Med Internet Res. 2026 May 12;28:e55941. doi: 10.2196/55941 (PMC13167057; doi:10.2196/55941)
Supplement: Multimedia Appendix 1 [file jmir-v28-e55941-s001.docx]

**Supplementary Material**

**Content**

Table S1 Subgroup analysis of maternal women’ preferences for smart bracelets according to income 2

Table S2 Subgroup analysis of maternal women’ preferences for smart bracelets according to occupation 3

Table S3 Subgroup analysis of maternal women’ preferences for smart bracelets according to pregnancy period (early and middle pregnancy) 4

Table S4 Subgroup analysis of maternal women’ preferences for smart bracelets according to pregnancy period (late pregnancy and postpartum) 5

Table S5 Subgroup analysis of maternal women’ preferences for smart bracelets according to whether or not for the first childbirth 6

Table S6 Subgroup analysis of maternal women’ preferences for smart bracelets according to whether or not have pregnancy complications 7

Table S7 Subgroup analysis of maternal women’ preferences for smart bracelets according to age 8

Table S1 Subgroup analysis of maternal women’ preferences for smart bracelets according to income

| **Attribute and levels** | **≤4000 (n=102)** | | **4001-6000 (n=170)** | | **≥6001 (n=192)** | |
| --- | --- | --- | --- | --- | --- | --- |
|  | **β（SE）** | **WTP** | **β（SE）** | **WTP** | **β（SE）** | **WTP** |
| cost | -0.000 (0.000) * | — | -0.000 (0.000) | — | -0.000 (0.000) |  |
| Hospital background monitoring management |  |  |  |  |  |  |
| No | Ref |  |  |  |  |  |
| Yes | 0.187 (0.229) | 389.623 | 0.400 (0.189) * | 1573.130 | 0.229 (0.158) | 1612.846 |
| Main function |  |  |  |  |  |  |
| Activity tracking | Ref |  |  |  |  |  |
| Sleep quality | 0.881 (0.158) *** | 1838.298 | 0.622 (0.122) *** | 2447.175 | 0.862 (0.117) *** | 6057.699 |
| Vital sign monitoring | 0.985 (0.238) *** | 2054.576 | 1.234 (0.189) *** | 4851.172 | 1.409 (0.185) *** | 9904.074 |
| Fetal heart monitoring | 1.227 (0.188) *** | 2559.351 | 1.073 (0.141) *** | 4218.230 | 1.552 (0.144) *** | 10912.899 |
| Privacy protection |  |  |  |  |  |  |
| No special protection | Ref |  |  |  |  |  |
| Standard protection | 0.392 (0.177) * | 817.850 | 0.165 (0.136) | 648.060 | 0.152 (0.134) | 1069.420 |
| High-level protection | 0.584 (0.222) ** | 1218.195 | 0.639 (0.168) *** | 2510.808 | 0.432 (0.166) ** | 3034.969 |
| Ease of use |  |  |  |  |  |  |
| Very difficult to use | Ref |  |  |  |  |  |
| Relatively difficult to use | 0.690 (0.255) ** | 1440.615 | 0.652 (0.191) ** | 2562.231 | 0.692 (0.186) *** | 4867.007 |
| Very easy to use | 0.628 (0.283) * | 1310.262 | 0.785 (0.217) *** | 3086.975 | 0.725 (0.210) ** | 5098.095 |
| Relatively easy to use | 1.012 (0.209) *** | 2112.600 | 0.895 (0.154) *** | 3517.957 | 1.071 (0.152) *** | 7526.625 |
| Frequency of monitoring report delivery to the smart bracelet |  |  |  |  |  |  |
| Once a week or less often | Ref |  |  |  |  |  |
| Once every two days | 0.794 (0.281) ** | 1657.443 | 0.584 (0.214) ** | 2297.221 | 0.428 (0.214) * | 3005.889 |
| Once a day | 0.957 (0.227) *** | 1996.970 | 0.859 (0.178) *** | 3377.200 | 0.848 (0.175) *** | 5962.055 |
| *P＜0.05  **P＜0.01  ***P＜0.001 | | | | | | |

Table S2 Subgroup analysis of maternal women’ preferences for smart bracelets according to occupation

| **Attribute and levels** | **Employed (n=353)** | | **No fixed occupation (Freelancer)(n=51)** | | **Unemployed or out of work (n=60)** | |
| --- | --- | --- | --- | --- | --- | --- |
|  | **β（SE）** | **WTP** | **β（SE）** | **WTP** | **β（SE）** | **WTP** |
| cost | -0.0001666 (0.0001136) | — | -0.0005283 (0.0003297) | — | -0.0006076 (0.0002948) * |  |
| Hospital background monitoring management |  |  |  |  |  |  |
| No | Ref |  |  |  |  |  |
| Yes | 0.226 (0.120) | 1355.210 | 0.885 (0.376) * | 1674.678 | 0.021 (0.325) | 33.943 |
| Main function |  |  |  |  |  |  |
| Activity tracking | Ref |  |  |  |  |  |
| Sleep quality | 0.779 (0.085) *** | 4676.624 | 0.396 (0.245) | 750.384 | 1.019 (0.222) *** | 1677.248 |
| Vital sign monitoring | 1.220 (0.130) *** | 7323.695 | 0.868 (0.356) * | 1643.161 | 1.753 (0.367) *** | 2885.119 |
| Fetal heart monitoring | 1.342 (0.101) *** | 8058.092 | 0.809 (0.281) ** | 1530.621 | 1.473 (0.252) *** | 2424.067 |
| Privacy protection |  |  |  |  |  |  |
| No special protection | Ref |  |  |  |  |  |
| Standard protection | 0.203 (0.096) * | 1221.149 | 0.485 (0.273) | 918.901 | 0.148 (0.241) | 244.302 |
| High-level protection | 0.514 (0.118) *** | 3085.083 | 1.372 (0.340) *** | 2597.462 | 0.125 (0.321) | 205.189 |
| Ease of use |  |  |  |  |  |  |
| Very difficult to use | Ref |  |  |  |  |  |
| Relatively difficult to use | 0.640 (0.132) *** | 3843.116 | 0.837 (0.405) * | 1584.427 | 0.742 (0.353) * | 1220.502 |
| Very easy to use | 0.658 (0.150) *** | 3951.900 | 1.414 (0.436) ** | 2676.214 | 0.573 (0.403) | 943.888 |
| Relatively easy to use | 0.968 (0.108) *** | 5809.533 | 1.053 (0.315) ** | 1993.210 | 1.025 (0.293) *** | 1687.239 |
| Frequency of monitoring report delivery to the smart bracelet |  |  |  |  |  |  |
| Once a week or less often | Ref |  |  |  |  |  |
| Once every two days | 0.550 (0.151) *** | 3299.520 | 0.030 (0.425) | 55.905 | 1.216 (0.403) ** | 2001.031 |
| Once a day | 0.841 (0.124) *** | 5048.346 | 0.396 (0.344) | 750.145 | 1.602 (0.336) *** | 2636.419 |
| *P＜0.05  **P＜0.01  ***P＜0.001 | | | | | | |

Table S3 Subgroup analysis of maternal women’ preferences for smart bracelets according to pregnancy period (early and middle pregnancy)

| **Attribute and levels** | **Early pregnancy (n=122)** | | **Mid-pregnancy (n=107)** | |
| --- | --- | --- | --- | --- |
|  | **β（SE）** | **WTP** | **β（SE）** | **WTP** |
| cost | -0.0005851 (0.0001963) ** | — | -0.0001094 (0.0002111) | — |
| Hospital background monitoring management |  |  |  |  |
| No | Ref |  |  |  |
| Yes | 0.340 (0.209) | 581.350 | 0.247 (0.245) | 2258.711 |
| Main function |  |  |  |  |
| Activity tracking | Ref |  |  |  |
| Sleep quality | 0.837 (0.141) *** | 1430.070 | 0.763 (0.159) *** | 6976.891 |
| Vital sign monitoring | 1.231 (0.219) *** | 2103.216 | 1.288 (0.246) *** | 11774.216 |
| Fetal heart monitoring | 1.085 (0.168) *** | 1854.717 | 1.338 (0.182) *** | 12226.078 |
| Privacy protection |  |  |  |  |
| No special protection | Ref |  |  |  |
| Standard protection | 0.134 (0.160) | 229.757 | 0.241 (0.176) | 2199.780 |
| High-level protection | 0.624 (0.204) ** | 1066.409 | 0.460 (0.217) * | 4205.345 |
| Ease of use |  |  |  |  |
| Very difficult to use | Ref |  |  |  |
| Relatively difficult to use | 0.875 (0.233) *** | 1494.538 | 0.609 (0.240) * | 5568.792 |
| Very easy to use | 0.900 (0.260) ** | 1538.641 | 0.611 (0.273) * | 5580.124 |
| Relatively easy to use | 1.125 (0.188) *** | 1923.243 | 0.998 (0.204) *** | 9117.059 |
| Frequency of monitoring report delivery to the smart bracelet |  |  |  |  |
| Once a week or less often | Ref |  |  |  |
| Once every two days | 0.519 (0.255) * | 887.508 | 0.633 (0.275) * | 5786.816 |
| Once a day | 0.826 (0.206) *** | 1412.426 | 0.874 (0.231) *** | 7983.197 |
| *P＜0.05  **P＜0.01  ***P＜0.001 | | | | |

Table S4 Subgroup analysis of maternal women’ preferences for smart bracelets according to pregnancy period (late pregnancy and postpartum)

| **Attribute and levels** | **Late pregnancy (n=110)** | | **Postpartum (n=125)** | |
| --- | --- | --- | --- | --- |
|  | **β（SE）** | **WTP** | **β（SE）** | **WTP** |
| cost | -0.0005734 (0.0002254) * | — | 0.0001276 (0.0001904) | — |
| Hospital background monitoring management |  |  |  |  |
| No | Ref |  |  |  |
| Yes | 0.174 (0.236) | 303.730 | 0.194 (0.187) | -1519.614 |
| Main function |  |  |  |  |
| Activity tracking | Ref |  |  |  |
| Sleep quality | 0.581 (0.162) *** | 1014.003 | 0.878 (0.148) *** | -6879.192 |
| Vital sign monitoring | 1.051 (0.243) *** | 1832.623 | 1.363 (0.223) *** | -10682.523 |
| Fetal heart monitoring | 1.601 (0.207) *** | 2792.522 | 1.272 (0.162) *** | -9974.017 |
| Privacy protection |  |  |  |  |
| No special protection | Ref |  |  |  |
| Standard protection | 0.445 (0.187) * | 775.772 | 0.154 (0.162) | -1205.610 |
| High-level protection | 0.849 (0.241) *** | 1479.897 | 0.384 (0.195) * | -3006.375 |
| Ease of use |  |  |  |  |
| Very difficult to use | Ref |  |  |  |
| Relatively difficult to use | 0.780 (0.292) ** | 1360.364 | 0.488 (0.212) * | -3824.029 |
| Very easy to use | 1.108 (0.308) *** | 1932.990 | 0.437 (0.245) | -3428.517 |
| Relatively easy to use | 1.078 (0.208) *** | 1879.451 | 0.820 (0.184) *** | -6429.785 |
| Frequency of monitoring report delivery to the smart bracelet |  |  |  |  |
| Once a week or less often | Ref |  |  |  |
| Once every two days | 0.630 (0.293) * | 1097.908 | 0.497 (0.256) | -3898.267 |
| Once a day | 1.009 (0.238) *** | 1758.725 | 0.843 (0.212) *** | -6605.000 |
| *P＜0.05  **P＜0.01  ***P＜0.001 | | | | |

Table S5 Subgroup analysis of maternal women’ preferences for smart bracelets according to whether or not for the first childbirth

| **Attribute and levels** | **No First childbirth(n=130)** | | **First childbirth Yes(n=334)** | |
| --- | --- | --- | --- | --- |
|  | **β（SE）** | **WTP** | **β（SE）** | **WTP** |
| cost | -0.0005258 (0.000184) ** | — | -0.0001461 (0.0001197) | — |
| Hospital background monitoring management |  |  |  |  |
| No | Ref |  |  |  |
| Yes | 0.143 (0.193) | 271.433 | 0.271 (0.127) * | 1854.654 |
| Main function |  |  |  |  |
| Activity tracking | Ref |  |  |  |
| Sleep quality | 0.941 (0.133) *** | 1789.723 | 0.703 (0.089) *** | 4812.265 |
| Vital sign monitoring | 1.272 (0.203) *** | 2420.006 | 1.235 (0.139) *** | 8450.936 |
| Fetal heart monitoring | 1.278 (0.161) *** | 2431.585 | 1.292 (0.105) *** | 8842.245 |
| Privacy protection |  |  |  |  |
| No special protection | Ref |  |  |  |
| Standard protection | 0.295 (0.153) | 560.325 | 0.179 (0.100) | 1228.701 |
| High-level protection | 0.577 (0.189) ** | 1097.774 | 0.524 (0.125) *** | 3586.757 |
| Ease of use |  |  |  |  |
| Very difficult to use | Ref |  |  |  |
| Relatively difficult to use | 0.682 (0.219) ** | 1297.765 | 0.663 (0.140) *** | 4537.650 |
| Very easy to use | 0.679 (0.244) ** | 1290.691 | 0.737 (0.158) *** | 5041.530 |
| Relatively easy to use | 0.987 (0.178) *** | 1877.805 | 0.991 (0.113) *** | 6781.225 |
| Frequency of monitoring report delivery to the smart bracelet |  |  |  |  |
| Once a week or less often | Ref |  |  |  |
| Once every two days | 0.485 (0.242) * | 921.715 | 0.615 (0.159) *** | 4208.252 |
| Once a day | 0.778 (0.196) *** | 1479.116 | 0.929 (0.131) *** | 6360.930 |
| *P＜0.05  **P＜0.01  ***P＜0.001 | | | | |

Table S6 Subgroup analysis of maternal women’ preferences for smart bracelets according to whether or not have pregnancy complications

| **Attribute and levels** | No Complications(n=390) | | Complications yes(n=74) | |
| --- | --- | --- | --- | --- |
|  | **β（SE）** | **WTP** | **β（SE）** | **WTP** |
| cost | -0.0002548 (0.0001086) * | — | -0.0002896 (0.0002572) | — |
| Hospital background monitoring management |  |  |  |  |
| No | Ref |  |  |  |
| Yes | 0.239 (0.114) * | 939.807 | 0.197 (0.268) | 681.616 |
| Main function |  |  |  |  |
| Activity tracking | Ref |  |  |  |
| Sleep quality | 0.739 (0.080) *** | 2899.084 | 0.938 (0.190) *** | 3238.704 |
| Vital sign monitoring | 1.250 (0.125) *** | 4905.814 | 1.195 (0.290) *** | 4124.954 |
| Fetal heart monitoring | 1.288 (0.092) *** | 5056.214 | 1.245 (0.259) *** | 4299.626 |
| Privacy protection |  |  |  |  |
| No special protection | Ref |  |  |  |
| Standard protection | 0.202 (0.090) * | 793.862 | 0.293 (0.219) | 1011.471 |
| High-level protection | 0.520 (0.112) *** | 2040.583 | 0.641 (0.269) * | 2214.995 |
| Ease of use |  |  |  |  |
| Very difficult to use | Ref |  |  |  |
| Relatively difficult to use | 0.690 (0.127) *** | 2709.838 | 0.562 (0.307) | 1941.263 |
| Very easy to use | 0.736 (0.143) *** | 2890.060 | 0.625 (0.354) | 2157.638 |
| Relatively easy to use | 0.988 (0.102) *** | 3878.098 | 0.994 (0.256) *** | 3430.970 |
| Frequency of monitoring report delivery to the smart bracelet |  |  |  |  |
| Once a week or less often | Ref |  |  |  |
| Once every two days | 0.532 (0.143) *** | 2087.983 | 0.804 (0.353) * | 2777.250 |
| Once a day | 0.866 (0.118) *** | 3400.375 | 0.989 (0.287) ** | 3416.629 |
| *P＜0.05  **P＜0.01  ***P＜0.001 | | | | |

Table S7 Subgroup analysis of maternal women’ preferences for smart bracelets according to age

| **Attribute and levels** | **Age ≤ 30 (n=228)** | | **Age ＞ 30 (n=236)** | |
| --- | --- | --- | --- | --- |
|  | **β（SE）** | **WTP** | **β（SE）** | **WTP** |
| cost | -0.0003556 (0.0001473) * | — | -0.0001628 (0.0001368) | — |
| Hospital background monitoring management |  |  |  |  |
| No | Ref |  |  |  |
| Yes | 0.415 (0.159) ** | 1167.885 | 0.103 (0.142) | 632.709 |
| Main function |  |  |  |  |
| Activity tracking | Ref |  |  |  |
| Sleep quality | 0.687 (0.109) *** | 1931.976 | 0.846 (0.101) *** | 5196.879 |
| Vital sign monitoring | 1.292 (0.171) *** | 3633.222 | 1.212 (0.156) *** | 7441.283 |
| Fetal heart monitoring | 1.234 (0.127) *** | 3469.057 | 1.339 (0.121) *** | 8224.679 |
| Privacy protection |  |  |  |  |
| No special protection | Ref |  |  |  |
| Standard protection | 0.197 (0.122) | 555.144 | 0.240 (0.115) * | 1471.784 |
| High-level protection | 0.669 (0.155) *** | 1881.899 | 0.427 (0.141) ** | 2623.321 |
| Ease of use |  |  |  |  |
| Very difficult to use | Ref |  |  |  |
| Relatively difficult to use | 0.719 (0.176) *** | 2022.328 | 0.634 (0.158) *** | 3894.737 |
| Very easy to use | 0.783 (0.197) *** | 2201.238 | 0.659 (0.179) *** | 4045.840 |
| Relatively easy to use | 1.020 (0.140) *** | 2868.713 | 0.967 (0.130) *** | 5940.336 |
| Frequency of monitoring report delivery to the smart bracelet |  |  |  |  |
| Once a week or less often | Ref |  |  |  |
| Once every two days | 0.627 (0.197) ** | 1762.833 | 0.519 (0.180) ** | 3186.398 |
| Once a day | 0.922 (0.162) *** | 2593.759 | 0.845 (0.148) *** | 5187.890 |
| *P＜0.05  **P＜0.01  ***P＜0.001 | | | | |
